# Slope analysis for the prediction of fluid responsiveness by a stepwise PEEP elevation recruitment maneuver in mechanically ventilated patients

**DOI:** 10.1186/s12871-021-01544-x

**Published:** 2022-01-03

**Authors:** Sylvain Vallier, Jean-Baptiste Bouchet, Olivier Desebbe, Camille Francou, Darren Raphael, Bernard Tardy, Laurent Gergele, Jérôme Morel

**Affiliations:** 1 Department of Anesthesiology and Intensive Care, Elsan Alpes-Belledonne Clinic, Grenoble, France; 2 Department of Anesthesiology and Intensive Care, Etienne University Hospital, Jean-Monnet University, SaintSaint-Etienne, France; 3 Department of Anesthesiology and Intensive Care, Ramsay Sante Sauvegarde Clinic, Lyon, France; 4 Department of Anesthesiology & Perioperative Care, University of California, Irvine, USA; 5 Centre d’Investigation Clinique - CIC 1408, Etienne University Hospital, Jean-Monnet University, SaintSaint-Etienne, France; 6 Department of Anesthesiology and Intensive Care, Ramsay Sante HPL Clinic, Saint-Etienne, France

**Keywords:** Lung recruitment maneuver, Fluid responsiveness, Central venous pressure, Pulse pressure, Hemodynamics, Mechanical ventilation

## Abstract

**Objective:**

Assessment of fluid responsiveness is problematic in intensive care unit patients. Lung recruitment maneuvers (LRM) can be used as a functional test to predict fluid responsiveness. We propose a new test to predict fluid responsiveness in mechanically ventilated patients by analyzing the variations in central venous pressure (CVP) and systemic arterial parameters during a prolonged sigh breath LRM without the use of a cardiac output measuring device.

**Design:**

Prospective observational cohort study.

**Setting:**

Intensive Care Unit, Saint-Etienne University Central Hospital.

**Patients:**

Patients under mechanical ventilation, equipped with invasive arterial blood pressure, CVP, pulse contour analysis (PICCO™), requiring volume expansion, with no right ventricular dysfunction.

Interventions.

None.

**Measurements and main results:**

CVP, systemic arterial parameters and stroke volume (SV) were recorded during prolonged LRM followed by a 500 mL fluid expansion to asses fluid responsiveness. 25 patients were screened and 18 patients analyzed. 9 patients were responders to volume expansion and 9 were not. Evaluation of hemodynamic parameters suggested the use of a linear regression model. Slopes for systolic arterial pressure, pulse pressure (PP), CVP and SV were all significantly different between responders and non-responders during the pressure increase phase of LRM (STEP-UP) (*p* = 0.022, *p* = 0.014, *p* = 0.006 and *p* = 0.038, respectively). PP and CVP slopes during STEP-UP were strongly predictive of fluid responsiveness with an AUC of 0.926 (95% CI, 0.78 to 1.00), sensitivity = 100%, specificity = 89% and an AUC = 0.901 (95% CI, 0.76 to 1.00), sensibility = 78%, specificity = 100%, respectively. Combining sensitivity of PP and specificity of CVP, prediction of fluid responsiveness can be achieved with 100% sensitivity and 100% specificity (AUC = 0.96; 95% CI, 0.90 to 1.00). One patient showed inconclusive values using the grey zone approach (5.5%).

**Conclusions:**

In patients under mechanical ventilation with no right heart dysfunction, the association of PP and CVP slope analysis during a prolonged sigh breath LRM seems to offer a very promising method for prediction of fluid responsiveness without the use and associated cost of a cardiac output measurement device.

**Trial registration:**

NCT04304521, IRBN902018/CHUSTE. Registered 11 March 2020, Fluid responsiveness predicted by a stepwise PEEP elevation recruitment maneuver in mechanically ventilated patients (STEP-PEEP)

## Introduction

Hemodynamic and fluid optimization during the perioperative period has been shown to reduce postoperative morbidity [1]. Unfortunately, the assessment of preload and determination of whether the patient will be fluid responsive has proved challenging. Static preload indices such as central venous pressure are not sufficient to assess fluid responsiveness [2]*,* whereas dynamic preload indices such as pulse pressure variation (PPV) and stroke volume (SV) variation have been used successfully [3]. However, such indices suffer from several limitations and should be used only under specific conditions [4]. Alternative dynamic methods of assessment such as respiratory systolic variation test (RSVT) [5] and lung recruitment maneuvers (LRM) have been developed [6]. LRM can be used to reopen or prevent atelectasis during mechanical ventilation in order to decrease respiratory complications [7]. LRM induce a transient increase in intra-thoracic pressure and a decrease in venous return, leading to a decrease in left ventricular end-diastolic area and stroke volume [8, 9]. Several studies have shown that the PEEP-induced decrease in stroke volume is related to pre-existing preload responsiveness [10, 11]. A few studies have also shown that LRM can be used as a functional test to predict fluid responsiveness [12–15]. However, monitoring stroke volume during LRM to assess fluid responsiveness is costly, and cardiac output devices may not be reliable [16]. In this context, central venous pressure (CVP) or systemic arterial monitoring represents a cost effective and readily available alternative for predicting fluid responsiveness during major surgery.

LRM can be performed using a prolonged sigh breath or stepwise increase in PEEP and airway inspiratory pressure with a constant drive pressure [17, 18]. These maneuvers have been described for 2 to 4-min periods. Prolonged LRM leads to a smaller increase in transpulmonary pressure for a longer period of time and improves lung aeration as effectively as sustained inflation, with less risk of hemodynamic compromise or hyperinflation. A recent study has specifically evaluated the hemodynamic response in this context [19]*.*

The aims of the current study were (1) to predict fluid responsiveness using changes in hemodynamic measurements during a stepwise increase in PEEP LRM in mechanically ventilated patients, (2) to identify the best criteria for predicting fluid responsiveness among changes in systolic arterial pressure (SAP), mean arterial pressure (MAP), diastolic arterial pressure (DAP), pulse pressure (PP) and central venous pressure (CVP), and (3) to compare the ability of these criteria with pulse pressure variation (PPV) to predict fluid responsiveness.

## Material and methods

We conducted a prospective study in the 23-bed intensive care unit (ICU) at Saint-Etienne University Medical Center, France, between December 2019 and December 2020. The study protocol was approved by the hospital’s ethics committee (Ethics Committee, Department of Anesthesiology, Saint-Etienne University Central Hospital, institutional Review Board IORG0007394, Protocol number IRBN902018/CHUSTE). All methods were performed in accordance with the relevant guidelines and regulations. Written informed consent was obtained for all study patients or relatives if indicated. Oral consent was obtained and reported in the medical record. Inclusion criteria were as follows: invasive arterial blood pressure and pulse contour analysis (PICCO system, Pulsion Medical Systems SE, Feldkirchen, Germany) for cardiac output measurement, central venous pressure monitoring, use of protective mechanical ventilation, age greater than 18 years and indication for volume expansion. A transthoracic echocardiography was performed on all patients prior to inclusion. Non-inclusion criteria were right ventricular dysfunction, significant valvulopathy, ejection fraction less than 50%, arrhythmia or presence of spontaneous breathing cycles. The inclusion time was at the start of the LRM. Exclusion criteria were: LRM not completed, absence of fluid expansion performed after LRM and patient decline for enrollment after reawakening.

### Sedation & monitoring

Each patient was monitored with pulse oximetry and a 5-lead EKG. Central venous pressure was measured continuously for all patients. All patients were equipped with a Transcardiopulmonary Thermodilution-Calibrated Arterial Waveform Analysis (PICCO system) inserted into a femoral artery. Pressure transducers were placed at the level of the mid-axillary line throughout the study protocol. All patients were intubated and ventilated using a volume-controlled mode. Sedation was maintained with propofol and/or midazolam in combination with either sufentanil or remifentanil. Neuromuscular blockade was not systematically used. The tidal volume (TV) was set by the clinician to the ideal body weight to obtain [6–8] mL/kg and the ventilatory rate was set in order to maintain arterial CO2 tension between 35 and 45 mmHg.

### Lung recruitment maneuver

**Fig. 1:**
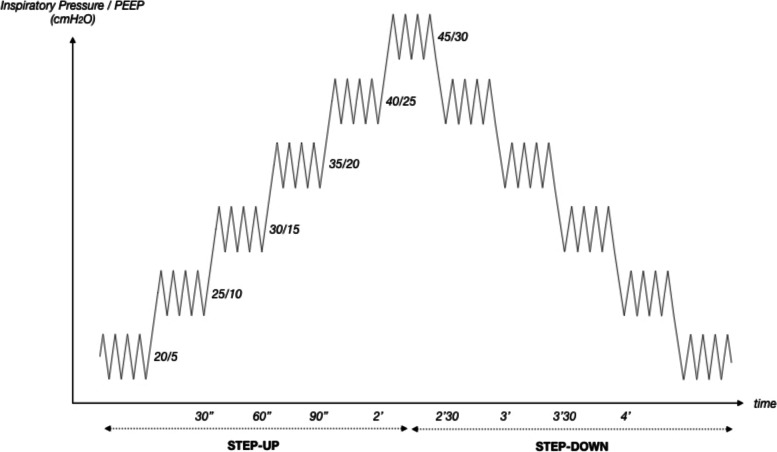
Schematic representation of STEP-PEEP lung recruitment maneuver

LRM were performed using a stepwise increase in PEEP and airway inspiratory pressure with the same drive pressure (15 cmH2O), as described in the literature [16, 17, 19]. The LRM consisted of a 5 cmH_2_O PEEP and inspiratory pressure increase every 30 s. The baseline was 5 cmH_2_O PEEP and 20 cmH_2_O inspiratory pressure for all patients. The maximum pressure level reached was 30 cmH_2_O PEEP and 45 cmH2O inspiratory pressure. PEEP de-escalation was performed following the same pattern (Fig. 1). After LRM, ventilatory settings were set back to initial patient settings. We defined the increasing levels of pressure as “STEP-UP” and the decreasing levels of pressure as “STEP-DOWN”. LRM were stopped if severe arterial hypotension (systolic arterial pressure less than 70 mmHg) or severe hypoxemia (SpO2 < 80%) was observed [18]. All patients considered for analysis received a fluid expansion after LRM. Fluid challenge was standardized and consisted of Plasmalyte infusion of 500 ml over 10 min.

### Data collection

We recorded demographic data including weight, age, gender, Simplified Acute Physiology Score (SAPS II), medical history, criteria of admission to ICU and comorbidities. Respiratory parameters (tidal volume, respiratory rate, insufflation pressure [peak], plateau pressure, level of PEEP), hemodynamic parameters (SAP, DAP, MAP), pulse pressure (PP = SAP-DAP), CVP, heart rate, PPV, and PICCO data (stroke volume and cardiac output (CO)) were also recorded. Baseline PPV was displayed on Phillips monitors. Pharmacological data (vasoactive infusions) and biological data (lactate) were recorded.

### Study protocol

When a patient met the inclusion criteria, the investigating physicians collected a set of demographic, ventilatory and hemodynamic data. Central venous catheter were inserted in right internal jugular vein, tip position was controlled to be in the right atrium using chest X-ray before inclusion. Zero of CVP was made at 0 degrees inclination. A transcardiopulmonary thermodilution was performed. Three injections of 20 mL cold fluid bolus were used for SV and CO calculations at baseline (mean of the three bolus). The LRM was then performed following the STEP-PEEP pattern as described above. A video of the hemodynamic monitoring was recorded during the LRM, with the clinician announcing the time and pressure level for each step. Hemodynamic values were later documented by pausing on the video during the two last seconds of each LRM step as announced by the physician. Next, a fluid expansion of Plasmalyte 500 mL over 10 min was performed. A second transcardiopulmonary thermodilution was done between 2 to 4 min after fluid expansion, using the same method. Responders (R) and Non-Responders (NR) were defined with regard to the change in SV (expressed as percentage) after fluid expansion. A fluid responder was defined as a 15% increase in SV after fluid expansion [20]*.*

### Statistics

A sample size of 18 patients (9 responders and 9 non-responders) was calculated to be sufficient to demonstrate that CVP and PP variations can predict fluid responsiveness with an area under curve (AUC) of 0.85, a power of 80% (beta risk = 0.2) and an alpha risk of 0.05. Data are expressed as mean (SD) or median (25^th^ to 75^th^). We used the Student T-test for continuous variables. Slope values were obtained using a linear regression calculation are expressed in degrees for the angle (α) between the horizontal axis and the linear regression curve calculated between the drop of pressure (mmHg) and the PEEP level (cmH2O). The threshold for statistical significance was set to *P* < 0.05. A receiver-operating characteristic (ROC) curve was drawn for αSAP, αMAP, αDAP, αPP and αCVP for STEP-UP and STEP-DOWN during LRM, respectively. We selected the threshold that gave the highest Youden index. We defined the grey zone for which inconclusive conclusions could not be obtained for values with a sensitivity lower than 90% or specificity lower than 90% according to Cannesson et al [21]. The method described by DeLong et al. was used to compare the areas under the ROC curve associated with the variables [22]. Statistical analyses were performed with XLSTAT software (version 2019.3.2).

## Results

### Patient characteristics

**Fig. 2:**
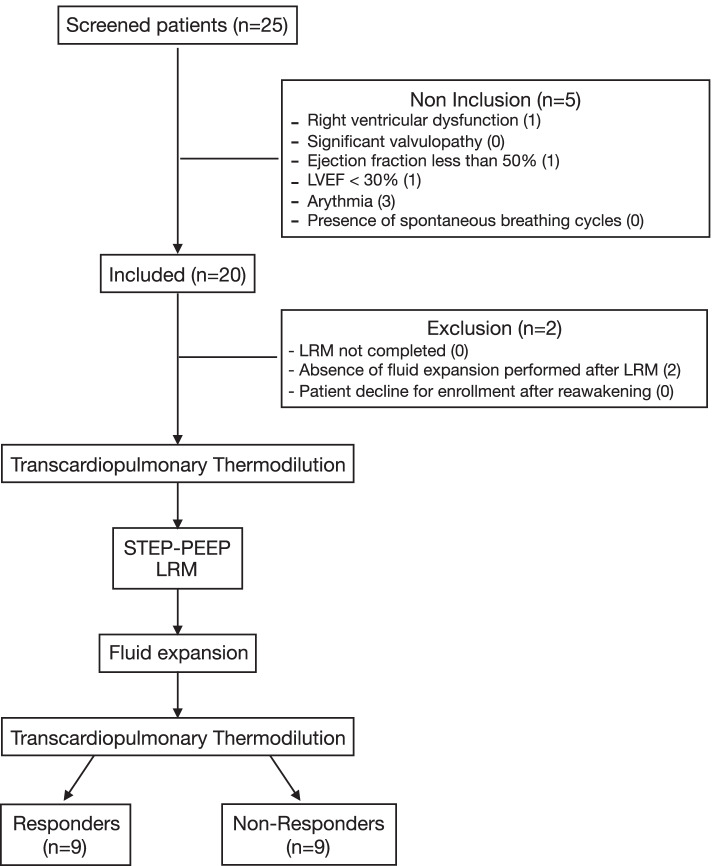
Study flow chart

A total of 25 nonconsecutive patients were screened. Five patients were not included due to right ventricular dysfunction (1 patient), ejection fraction less than 50% (1 patient) and presence of arrythmia (3 patients). Two patients were excluded due to absence of fluid expansion performed after LRM. A total of 18 patients were analyzed (Fig. 2). Nine patients (50%) were responders to volume expansion and nine were not. Patient main characteristics, hemodynamic, respiratory, pharmacological and biological variables in both Responders and Non-Responders are shown in Table 1. The PPV value was not displayed by the Philips monitor for 8 patients. Mean MAP during Pinsp 45 cmH_2_0 was 58.8 ± 4.15 mmHg.

**Table 1:** Patient demographic data, hemodynamic, respiratory, pharmacological and biological variables at baseline in Responders (*n* = 9) and Non-Responders (*n* = 9 patients)

	**Overall population**	**Responders**	**Non-Responders**	**p value**
	***N* = 18**	***N* = 9**	***N* = 9**	
Age (mean SD), yr	60 (15)	66 (7)	57 (18)	
Gender (M/F)	10/8	5/4	5/4	
BMI (mean SD), kg.m-2	29 (9)	29 (6)	29 (12)	
Ideal body weight (mean SD), kg	66 (14)	63 (11)	66 (17)	
SAPS 2 (mean SD)	61 (22)	69 (22)	57 (21)	
ICU admission criteria				
Septic shock	4	3	1	
Cardiac failure	7	3	4	
Respiratory failure	4	2	2	
Hemorrhagic shock	1	1	0	
Polytrauma	1	0	0	
Cranio-cerebral trauma	1	0	1	
Comorbidities				
Arterial hypertension	8	6	2	
Diabetes	4	2	2	
Coronary artery disease	1	1	0	
Hemodynamic parameters				
Mean arterial pressure (mean SD), mmHg	73 (9)	70 (10)	76 (7)	0.110
Heart Rate (mean SD), HR/min	90 (18)	91 (23)	87 (11)	0.813
Stroke volume (mean SD), mL	62 (26)	53 (20)	64 (29)	0.177
Cardiac output (mean SD), L/min	5.3 (2.2)	4.5 (1.0)	5.5 (2.7)	0.150
PPV (mean SD), %	10 (8)	14 (10)	6 (2)	0.034
Central Venous Pressure (mean SD), mmHg	10 (4)	9 (3)	10 (4)	0.238
Respiratory parameters				
Tidal volume (mean SD), mL	436 (62)	452 (55)	405 (68)	0.288
Respiratory Rate (mean SD), RR/min	20 (5)	18 (4)	22 (6)	0.218
Positive end expiratory pressure (mean SD), cmH2O	10 (3)	9 (2)	10 (3)	0.671
Plateau pressure (mean SD), cmH2O	22 (6)	19 (3)	23 (8)	0.099
Static pulmonary compliance (mean SD), mL/cmH2O	42 (15)	48 (11)	37 (15)	0.065
P/F (mean SD)	236 (97)	224 (104)	242 (97)	0.679
Neuromuscular blockade, n	9	4	5	
Pharmacological parameters				
Norepinephrine (mean SD), ug/kg/min	0.50 (0.64)	0.78 (0.76)	0.24 (0.35)	0.031
Dobutamine (mean SD), ug/kg/min	2.06 (3.37)	1.44 (2.96)	2.67 (3.81)	0.394
Biological parameters				
Lactates (mean SD), mmol/l	2.2 (1.5)	2.7 (2.1)	2.0 (0.8)	0.215

The baseline norepinephrine concentration was higher in the Responders group (R) than in the Non-Responders (NR) group (0.78 ug/kg/min vs 0.24 ug/kg/min, respectively; p = 0.031). Baseline PPV was higher in the R than in the NR group (14% vs 6%, respectively; p = 0.034). R and NR did not differ for baseline values of SV, CO, MAP, CVP or lactate level. Static pulmonary compliance was not statistically different between R and NR.

### Prediction of fluid responsiveness

**Fig. 3:**
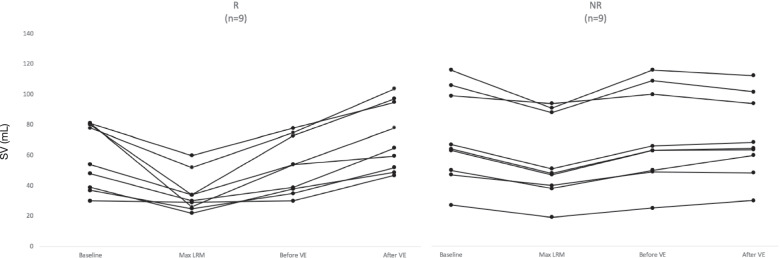
Representation of SV (mL) values for each step: baseline, maximum pressure level during LRM, before VE and after VE

SV values for baseline, maximum lung recruitment pressure, and before and after volume expansion are represented in Fig. 3.

**Fig. 4:**
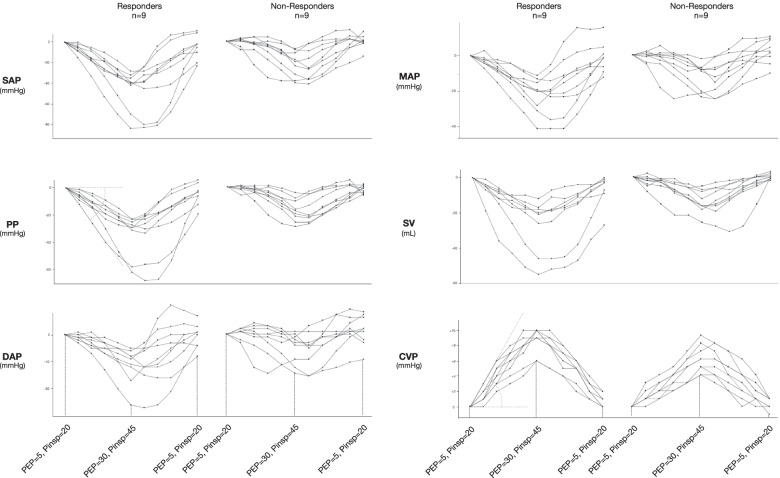
Line graphs showing the relationship between PEEP and change in pressure through the stepwise lung recruitment maneuver and example of alpha angle calculation between the X axis and the regression interpolation line

Six data points were available for each parameter for STEP-UP and STEP-DOWN. Figure 4 shows individual variations in hemodynamic parameters during LRM. STEP-UP LRM induced a decrease of SAP, PP, DAP, MAP, and SV and an increase of CVP. Fluid Responders demonstrated a greater decrease of SAP, PP, DAP, MAP, and SV as well as a greater increase of CVP compared to Non-Responders.

Evaluation of all hemodynamic variables suggested the use of a linear regression model, especially during STEP-UP LRM. Slope calculations are shown in Table 2. Slopes are reported as αSAP, αDAP and αMAP for systolic, diastolic and mean arterial pressure, αPP for pulse pressure, αCVP for central venous pressure and αSV for stroke volume.

**Table 2:** Slopes (degrees) for systolic arterial pressure (αSAP), pulse pressure (αPP), diastolic arterial pressure (αDAP), mean arterial pressure (αMAP), stroke volume (αSV) and central venous pressure (αCVP) for STEP-UP and STEP-DOWN during lung recruitment maneuver

	**STEP-UP**			**STEP-DOWN**		
	Responders	Non-Responders	p value	Responders	Non-Responders	p value
	***N* = 9**	***N* = 9**		***N* = 9**	***N* = 9**	
**α SAP (SD)**	-59.4° (5.79)	-37.5° (13.1)	0.022	57.1° (8.25)	40.8° (12.6)	0.034
**α PP (SD)**	-51.9° (7.24)	-31.6° (10.7)	0.014	48.8° (8.26)	31.8° (10.6)	0.010
**α DAP (SD)**	-23.6° (8.77)	-11.4° (10.0)	0.126	24.3° (9.44)	18.5° (10.1)	0.528
**α MAP (SD)**	-40.3° (8.50)	-24.2° (11.9)	0.089	39.6° (9.51)	30.1° (11.5)	0.228
**α SV (SD)**	-43.5° (9.65)	-27.4° (8.64)	0.038	25.9° (20.1)	29.0° (9.60)	0.837
**α CVP (SD)**	19.8° (2.66)	13.1° (3.03)	0.006	-18.2° (2.05)	-13.4° (2.60)	0.003

Slope calculations showed greater absolute values for the Responder compared to the Non-Responder group for STEP-UP and STEP-DOWN LRM (Table 2). The ability of αSAP, αPP, αDAP, αMAP, αSV and αCVP to predict fluid responsiveness and the results of AUC analysis are shown in Table 3. The best predictive variables for fluid responsiveness during LRM were αPP and αCVP during STEP-UP, with Youden indices of 0.888 and 0.777 respectively.

**Table 3:** Diagnostic performance of slopes for systolic arterial pressure (αSAP), pulse pressure (αPP), diastolic arterial pressure (αDAP), mean arterial pressure (αMAP), stroke volume (αSV), central venous pressure (αCVP) and relative variations from baseline of systolic arterial pressure (∆SAP), pulse pressure (∆PP), diastolic arterial pressure (∆DAP), mean arterial pressure (∆MAP), stroke volume (∆SV) and central venous pressure (∆CVP) between baseline (PEEP = 5, Inspiratory Pressure = 20cmH2O) and maximum pressure level (PEEP = 30, Inspiratory Pressure = 45cmH2O) to predict fluid responsiveness during STEP-UP and STEP-DOWN lung recruitment maneuver

**STEP-UP**	**αSAP**	**αPP**	**αDAP**	**αMAP**	**αSV**	**αCVP**
Cut-off value (degrees)	-47.8°	-42.8°	-10.1°	-20.1°	-34.2°	20.1°
ROC AUC	0.864	0.926	0.765	0.777	0.854	0.901
Sensitivity	1	1	1	1	0.875	0.777
Specificity	0.666	0.888	0.666	0.666	0.777	1
Positive predictive value	0.75	0.9	0.75	0.75	0.777	1
Negative predictive value	1	1	1	1	0.875	0.818
Youden index	0.666	0.888	0.666	0.666	0.653	0.777
Grey zone (degrees)	[47.8°-59.6°]	[42.8°-52.1°]	[10.1°-34.2°]	[20.1°-42.8°]	[24.3°-46.1°]	[13.8°-20.1°]
	**∆SAP**	**∆PP**	**∆DAP**	**∆MAP**	**∆SV**	**∆CVP**
Cut-off value (mmHg)	23	21	4	9	16	8
ROC AUC	0.901	0.920	0.777	0.790	0.753	0.883
Sensitivity	1	1	1	1	0.777	0.777
Specificity	0.666	0.777	0.666	0.666	0.777	0.888
Positive predictive value	0.75	0.818	0.75	0.75	0.777	0.875
Negative predictive value	1	1	1	1	0.777	0.8
Youden index	0.666	0.777	0.666	0.666	0.555	0.666
Grey zone (mmHg)	[23–39]	[21–28]	[4–14]	[9–23]	[0–25]	[5–9]
**STEP-DOWN**	**αSAP**	**αPP**	**αDAP**	**αMAP**	**αSV**	**αCVP**
Cut-off value (degrees)	55.4°	47.8°	20.4°	34.7°	43.2°	-14.1°
ROC AUC	0.777	0.815	0.666	0.685	0.666	0.877
Sensitivity	0.777	0.666	0.777	0.777	0.375	0.888
Specificity	0.777	0.888	0.666	0.666	1	0.777
Positive predictive value	0.777	0.857	0.7	0.7	1	0.8
Negative predictive value	0.777	0.727	0.75	0.75	0.643	0.875
Youden index	0.555	0.555	0.444	0.444	0.375	0.666
Grey zone (degrees)	[38.7°-67.8°]	[32.4°-52.3°]	[3.9°-36.0°]	[9.76°-50.6°]	[13.2°-43.2°]	[13.8°-20.1°]
	**∆SAP**	**∆PP**	**∆DAP**	**∆MAP**	**∆SV**	**∆CVP**
Cut-off value (mmHg)	32	21	8	17	15	5
ROC AUC	0.809	0.852	0.741	0.735	0.642	0.888
Sensitivity	0.777	0.777	0.777	0.777	0.666	1
Specificity	0.888	0.888	0.777	0.777	0.666	0.666
Positive predictive value	0.875	0.875	0.777	0.777	0.666	0.75
Negative predictive value	0.8	0.8	0.777	0.777	0.666	1
Youden index	0.666	0.666	0.555	0.555	0.333	0.666
Grey zone (mmHg)	[11–46]	[13–30]	[0–16]	[4–29]	[0–25]	[5–8]

αPP during STEP-UP was strongly predictive of fluid responsiveness with an AUC of 0.926 (95% CI, 0.78 to 1.00), and a sensitivity and a specificity of 100% and 89% respectively. Cut-off value was -42.8°. Inconclusive values ranged from -42.8° to -52.1° using the grey zone approach (35% of the patients).

αCVP during STEP-UP was also strongly predictive of fluid responsiveness with an AUC of 0.901 (95% CI, 0.76 to 1.00) and a sensitivity and a specificity of 78% and 100% respectively. The cut-off value was 20.1°. Inconclusive values ranged from 13.8° to 20.1° using the grey zone approach (44% of the patients).

By combining sensitivity of αPP and specificity of αCVP if both measures are available, taking for each angle the specificity value of αCVP and sensitivity value of αPP, fluid responsiveness prediction can be obtained with 100% sensitivity and 100% specificity during STEP-UP LRM (AUC = 0.96; 95% CI, 0.90 to 1.00). One patient (5.5%) showed inconclusive values using the grey zone approach (Fig. 4). Index combination has already been proposed to optimize the sensitivity and specificity of a parameter [23].

Absolute variations for SAP, PP, MAP, DAP, SV and CVP between baseline (PEEP = 5 mmHg, Inspiratory pressure = 20 mmHg) and maximum pressure level (PEEP = 30 mmHg, Inspiratory pressure = 45 mmHg) are reported as ∆SAP, ∆PP, ∆MAP, ∆DAP, ∆SV and ∆CVP. Their ability to predict fluid responsiveness is shown in Table 3.

**Fig. 5:**
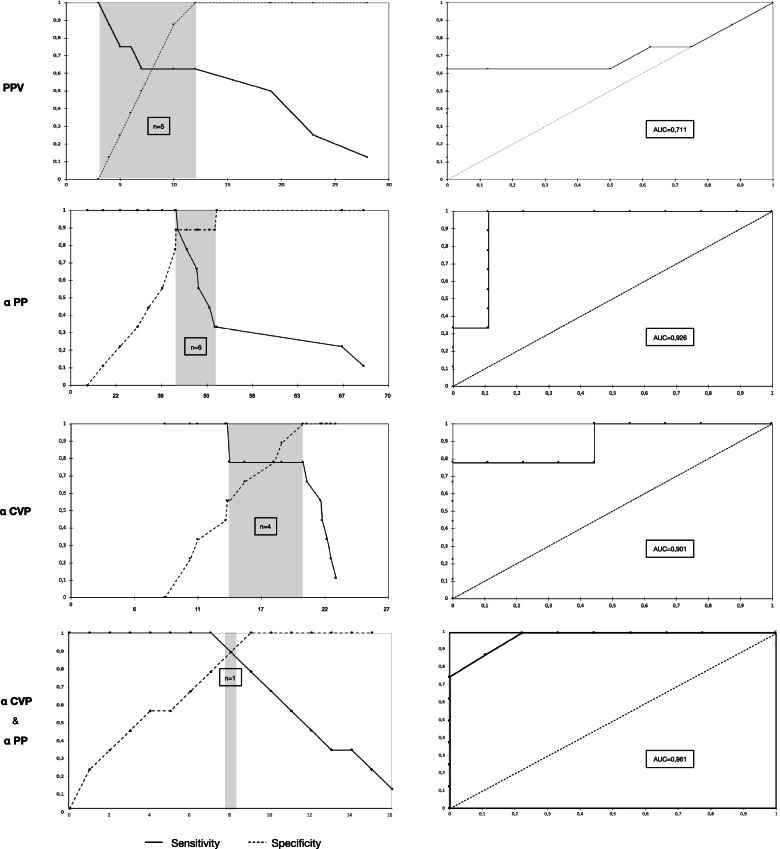
Sensitivity, specificity, grey zone (n =) and Receiver Operating Curves generated of pulse pressure variation (PPV) at baseline and slopes for changes in pulse pressure (αPP), central venous pressure (αCVP) and combination of αPP sensitivity & αCVP specificity during STEP-UP lung recruitment maneuver, with a view to discriminating between fluid expansion Responders and Non-Responders

PPV at baseline was available for 11 patients. A PPV of more than 12% before LRM predicted responders with an AUC of 0.711 (95% CI, 0.42 to 1.00) and a sensitivity and a specificity of 63% and 100% respectively. Inconclusive values ranged from 4 to 10% using the grey zone approach (45% of the patients) (Fig. 5).

## Discussion

In our study, results show that αCVP and αPP changes induced by a progressive lung pressure (STEP-PEEP) recruitment maneuver are the best hemodynamic parameters for the prediction of fluid responsiveness in mechanically ventilated patients in ICU, with or without neuromuscular blockade.

STEP-PEEP LRM offered the ability to evaluate the effects of LRM using a different approach from usual bi-level sustained insufflation LRM. Six data points were available for STEP-UP and STEP-DOWN for all parameters, following a linear evolution especially during STEP-UP, allowing a linear regression analysis. Absolute variations of PP and CVP between PEEP = 5cmH2O and PEEP = 30cmH2O during STEP-UP provide interesting results with respect to discrimination of fluid responders and non-responders. PPV was significantly different between Responders and Non-Responders at baseline but showed inferior statistical values compared to slope analysis. PPV and CVP follow a linear evolution during STEP-UP LRM and there is a possibility that the LRM method for fluid responsiveness prediction could offer a similar statistical performance using lower pressure LRM.

During LRM, a transient increase in intra-pulmonary pressure is transmitted to the adjacent intra-thoracic compartments. This increase in intrathoracic pressure most impacts the right ventricle, however no patients in our study had right ventricular cardiac dysfunction as they were excluded. Preload and afterload of the right ventricle are also affected by high intra-thoracic pressure. High intra-thoracic pressure increases the right ventricular afterload and ejectional impedance [24]. These effects are particularly significant when preload is low [8, 23]. All patients in our study displayed a significant decrease in SV and systemic arterial pressure, and an increase in CVP during LRM. Hemodynamic changes were more important in the Responders group.

Cardio-pulmonary interactions are widely studied and used in clinical practice, especially with PPV analysis and more recently with LRM hemodynamic response analysis [12–14] or end expiratory occlusion test [25–27]*.* Pressure transmission from lung and pleural space to the heart and mediastinum can be affected by many physiological and physio-pathological conditions. Compliance loss in acute respiratory distress syndrome for patients in intensive care units or catecholamine use can also lead to changes in cardio-pulmonary interactions. Therefore, it is important for clinicians not to oversimplify this physiology and to take into account the characteristics of the underlying pathologies before making any decision based on the scientific literature.

PP slope calculation (αPP) after linear regression during STEP-PEEP showed excellent sensitivity. This can be explained by the fact that if a transient increase in intra-thoracic pressure does not impact systemic arterial pressure, the right ventricular preload is likely to be sufficient to avoid collapse. αPP variation below cut-off value during STEP-UP could therefore exclude fluid responsiveness with a 100% sensitivity in our study.

CVP slope calculation (αCVP) during STEP-LRM showed excellent specificity. This can be explained by the fact that if a transient increase in intra-thoracic pressure induces a significant variation in CVP, the right ventricular preload is likely to be low and easily affected external pressure. αCVP above cut-off value during STEP-UP can confirm fluid responsiveness with 100% specificity in our study.

Clinicians may therefore benefit from the sensitivity of αPP using only an arterial catheter, or the specificity of αCVP using a central venous catheter for fluid responsiveness prediction. For patients equipped with both, combination of αPP and αCVP during STEP-UP allows clinicians to predict fluid responsiveness with 100% sensitivity and specificity.

This study has several limitations. This pilot study was monocentric and 18 only patients have been studied. Statistical results are highly significant but further larger studies are needed to confirm our preliminary results. Next, SV during LRM was calculated with pulse contour analysis. Surprisingly, the SV results for absolute variation and angle analysis were inferior to results for SAP, PP and CVP. SV calculated from pulse contour analysis may have been imprecise due to increasing levels of pressure and variation of systemic vascular resistances from baseline initial transcardiopulmonary thermodilution. Of note, thermodilution is the method of choice for cardiac output measurement and was used before LRM, and after fluid challenge.

Baseline norepinephrine differed between the R and NR groups. Differences of norepinephrine dosage may have had an effect on the stressed and unstressed fluid volume, potentially leading to bias in interpretation of CVP for evaluation of fluid responsiveness.

All included patients required fluid expansion as determined by the clinician in charge, based on vasopressor requirements, echocardiographic evidence or clinical judgement. Half of the patients included were found to have received suboptimal fluid management, illustrating the need for a specific tool for evaluation of fluid responsiveness.

Calculation of the slope may be challenging at bedside but a calculation of the α angle according to the successive values of CVP and PP can easily be automated on a computer or smartphone.

All patients were not consecutive patients, introducing a potential selection bias. Before inclusion, all patients required a fluid expansion as determined by the clinician in charge based on vasopressor requirements, echocardiographic evidence or clinical judgement. Inclusions were conducted exactly the same way for all patients, Responder or Non-Responder status being determined offline only after fluid expansion. The fact that half of the included patients were Non-Responders shows that our current tools are not accurate enough to discriminate Responders and Non-Responders.

The use and format of LRM can be debated. High intrathoracic pressures can have harmful effects on patients without lung disease. The STEP-PEEP lung recruitment maneuver was chosen from the literature as it showed lesser increase in transpulmonary pressure for a longer period of time and improved lung aeration as effectively as sustained inflation does, with less risk of hemodynamic compromise and hyperinflation [17, 18]*.*

## Conclusion

In mechanically ventilated patients, a progressive STEP-PEEP lung recruitment maneuver could predict fluid responsiveness using the slope analysis of pulse pressure (αPP) and central venous pressure (αCVP) evolutions. αPP variation below cut-off value during STEP-UP can exclude fluid responsiveness. αCVP above cut-off value during STEP-UP can affirm fluid responsiveness. In this pilot study, the association of αPP and αCVP during STEP-UP recruitment maneuver provides a high sensitivity and high specificity and seems to offer a very promising method for fluid responsiveness prediction without the use and cost of a cardiac output measurement device.

## Data Availability

The datasets used and/or analyzed during the current study are available from the corresponding author on reasonable request.

## Abbreviations

LRM: Lung Recruitment Maneuver
CVP: Central Venous Pressure
SV: Stroke Volume
PP: Pulse Pressure
PPV: Pulse Pressure Variation
SAP: Systolic Arterial Pressure
MAP: Mean Arterial Pressure
DAP: Diastolic Arterial Pressure
ICU: Intensive Care Unit
TV: Tidal Volume
CO: Cardiac Output
R: Responders (to volume expansion)
NR: Non Resonders (to volume expansion)
αSAP: Angle calculation between X axis and SAP regression interpolation line
αMAP: Angle calculation between X axis and MAP regression interpolation line
αDAP: Angle calculation between X axis and DAP regression interpolation line
αPP: Angle calculation between X axis and PP regression interpolation line
αCVP: Angle calculation between X axis and CVP regression interpolation line
αSV: Angle calculation between X axis and SV regression interpolation line
SD: Standard deviation
AUC: Area Under Curve
∆SAP: Absolute variation for SAP
∆PP: Absolute variation for PP
∆DAP: Absolute variation for DAP
∆MAP: Absolute variation for MAP
∆CVP: Absolute variation for CVP
∆SV: Absolute variation for SV
